# Functional Differences Across Playing Roles in Volleyball: A Sensor-Based Assessment

**DOI:** 10.3390/jfmk11020238

**Published:** 2026-06-13

**Authors:** Juri Taborri, Mauro Strippoli, Luca Molinaro, Stefano Rossi

**Affiliations:** 1 Department of Economics, Engineering, Society and Business Organization, University of Tuscia, 01100 Viterbo, Italy; mauro.strippoli@studenti.unitus.it (M.S.); stefano.rossi@unitus.it (S.R.); 2 Department of Theoretical and Applied Science, eCampus University, 22060 Novedrate, Italy; luca.molinaro@uniecampus.it

**Keywords:** sensor-based protocol, role-specific functional assessment, mobility, posturography, jump analysis, volleyball

## Abstract

**Objectives**: Volleyball playing positions are associated with different functional demands. This study compared postural control, jump performance, and upper-limb mobility across playing roles in competitive male volleyball players. **Methods**: Fifty male volleyball players competing in the Italian Serie C championship were equally distributed across five roles: middle blockers (MB), liberos (LIB), opposite hitters (OH), setters (SET), and outside hitters (HIT). Using a wearable inertial sensor, athletes performed bipodalic balance tasks with eyes open and closed, dominant- and non-dominant-leg single-leg balance, squat jump (SJ), countermovement jump (CMJ), and bilateral upper-limb flexion and extension tests. **Results**: Significant role-related differences emerged in balance and jump performance. In bipodalic balance, the eyes-open condition showed a mixed pattern, with HIT displaying the largest ellipse area and SET showing the highest path-related values, whereas in the eyes-closed condition, HIT showed the highest values across all stabilometric parameters. In the single-leg stance, OH showed the largest postural excursions on the dominant side, while LIB stood out on the non-dominant side. In jump tests, MB showed the best vertical performance in both SJ and CMJ, whereas LIB and SET generally showed the lowest outputs. Temporal differences also emerged across roles. Upper-limb mobility was similar across roles in flexion, while extension showed a role-specific pattern, with SET displaying greater ROM than LIB, HIT, and OH. **Conclusions**: Volleyball roles are associated with distinct functional profiles in balance, jump mechanics, and upper-limb mobility. This integrated assessment may support more specific training, monitoring, and injury-prevention strategies.

## 1. Introduction

Volleyball is a high-intensity intermittent team sport characterized by repeated jumps, short accelerations and decelerations, multidirectional displacements, dives, lunges, and overhead ball actions performed within brief rallies and limited court space [1]. These demands are not evenly distributed across the team since each player occupies a specific role, such as setter, outside hitter, middle blocker, opposite hitter, or libero, and is exposed to distinct technical, tactical, and biomechanical demands [2]. Fundamental technical skills such as serve, reception/passing, setting, attacking/spiking, and blocking are therefore not expressed uniformly across players, but through role-dependent movement solutions shaped by the specific functions assigned to each position [3]. In this context, the assessment of selected functional characteristics such as jump performance, postural control, and upper-limb mobility may help clarify the neuromuscular and biomechanical capacities underlying role-specific volleyball performance. In particular, jump ability reflects the capacity to generate rapid vertical impulse for blocking and attacking actions, postural control supports landing stabilization, body-orientation regulation, and rapid repositioning, whereas upper-limb mobility contributes to the efficient execution of repeated overhead actions by facilitating shoulder positioning, segmental coordination, and ball control [4].

Role-specific differences in volleyball have already been documented, mainly considering anthropometric perspectives and match statistics [5,6]. Palao et al. analyzed 1454 male and 1452 female players from the Olympic Games and World Championships between 2000 and 2012 using players’ position, body height, body mass, BMI, spike reach, block reach, age, and team level [7]. They showed systematic positional differences, with middle blockers, outside and opposite hitters, presenting profiles more favorable to blocking and spiking, and setters and liberos showing characteristics more consistent with setting, reception, and digging. A second line of research has distinguished roles through video-match analysis, especially by examining the specific tactical tasks attached to setters, liberos, and attackers. Alfonso et al. analyzed six matches from the 2006 Women’s World Championship (24 sets, 670 setting sequences), finding that attack timing was the key variable of the setter’s tactical action [8]. Specifically, jump-sets performed from the ideal setting zone, together with middle-attacker simulation and anticipatory blocking behavior, tended to culminate in quick attacks, whereas non-ideal zones and less constrained block scenarios were associated with slower tempos. At the libero level, García-de-Alcaraz and Usero [6] analyzed 1597 libero actions from 49 sets (13 top-level women’s matches) and reported that liberos participated mainly in reception and digging, achieved their best performance in reception and setting, and showed reduced participation and worse digging performance toward the end of sets [9]. At the attacker level, Lima et al. examined 8541 rallies from 74 matches in the 2018 European Golden League and showed that technical actions and attack efficacy also varied according to playing position, confirming that role specialization emerges not only in body profile but also in actual game production [10].

Although match statistics and position-specific game analyses provide valuable information on how roles differ during competition, they offer only indirect insight into the functional capacities that underlie the execution of those demands. From this perspective, more recent studies have approached positional differences through wearable technologies and functional screening, usually quantifying generic movement patterns rather than the execution of the fundamental volleyball skills themselves. Lima et al. monitored seven elite male players across five official matches and analyzed 1599 jumps using an inertial measurement unit for jump height together with video analysis for jump type [11]. They found different frequencies and intensities of jumps across middle blockers, setters, and outside hitters, providing position-specific jump profiles in competition. A more explicitly functional approach was adopted by Agostini et al., who tested 16 volleyball players from four roles using rasterstereography, a single-leg balance task, and IMU-based countermovement jump and stiffness tests [12]. Despite this broader battery, the only significant between-role difference emerged in left-limb stiffness. From a postural point of view, Agostini et al. investigated postural sway in volleyball players and showed that balance behavior in this population reflects sport-specific adaptations rather than generic static stability [13]. Along the same line, Borzucka et al. examined postural control in top-level female volleyball players and reported that volleyball practice is associated with specific balance strategies, likely related to the repeated demands of landing stabilization, rapid repositioning, and body-orientation control during play [14]. Moving to the upper-limb mobility, Ozawa et al. analyzed the biomechanics of the volleyball overhead pass and showed that shoulder and upper-limb movement organization play a key role in controlling ball direction and execution efficiency [15]. Although these studies support the relevance of shoulder-related function in volleyball, the literature has more often focused on technical gesture analysis than on simple field-based mobility profiling across playing roles. As a result, it is still unclear whether upper-limb mobility itself shows role-dependent patterns comparable to those already reported for anthropometry, jump performance, and external load.

Taken together, the available literature indicates that volleyball roles differ across several performance-related domains, but most previous studies have examined these domains separately, with particular emphasis on anthropometry, jump performance, or match load [5]. A more integrated perspective combining jump performance, postural control, and upper-limb mobility may provide a broader description of the functional characteristics associated with each playing role and may help practitioners better interpret positional demands in competitive volleyball. Therefore, the aim of the present study was to characterize role-specific functional profiles in competitive volleyball players by comparing postural control, jump performance, and upper-limb mobility across playing positions.

## 2. Materials and Methods

### 2.1. Participants

The observational study involved 50 male competitive volleyball players from two distinct teams from Viterbo and Tivoli. Both teams competing in the Italian Serie C championship. Participants were between 16 and 27 years of age (with a mean age of 20.5 ± 2.9 years), with an average height of 1.87 ± 0.20 m and an average body mass of 72.1 ± 8.8 kg. Lower-limb dominance was identified using a spontaneous forward-step response test, where participants were gently perturbed from behind, and the limb naturally used to step forward and restore balance was considered the dominant leg. The participants were equally distributed among the five main volleyball roles: (i) setter (SET); (ii) outside hitter (HIT); (iii) opposite hitter (OH); (iv) middle blocker (MB); and (v) libero (LIB). All athletes had several years of experience in their specific role and were regularly engaged in team training and official competition. To ensure functional integrity during testing, athletes with either acute or chronic musculoskeletal conditions affecting the lower or upper limbs and/or the spine were excluded if these had limited regular training participation or could interfere with task execution during the 12 months preceding data collection. Testing was performed voluntarily during the team’s regular sporting activities at the usual indoor training. Participants were first fully informed about the study’s purpose and they were asked to sign a written informed consent in compliance with the ethical principles of the 1964 Declaration of Helsinki. The research protocol was approved by the Ethical Committee of the University of Tuscia on 22 December 2024 (approval no. 22122024).

Table 1 reports anthropometric data divided per role.

### 2.2. Experimental Setup and Protocol

The experimental protocol was designed to provide an objective and ecologically valid functional assessment of postural control, lower-limb explosive performance, and upper-limb mobility. All tests were performed in the athletes’ habitual training environment in order to reduce contextual bias and preserve task-specific motor behavior. Before experimental acquisition, each participant underwent a warm-up. Specifically, the warm-up lasted approximately 20 min and included low-intensity running, dynamic joint mobility exercises, volleyball-specific technical movements, and a series of submaximal jumps to prepare the athletes for the subsequent testing battery. Successively, participants completed a standardized battery of motor tasks in a random order, with recovery intervals between trials to minimize the effects of fatigue and maintain movement quality.

The assessment battery included four main tests: (i) bipodalic static balance with eyes open and eyes closed; (ii) right and left single-leg balance; (iii) vertical jump performance assessed through Squat Jump (SJ) and Countermovement Jump (CMJ); and (iv) upper-limb flexion and extension performed separately with the right and left arms. A recovery interval of approximately 30 s was provided between repetitions of the same task, whereas about 60 s of rest was allowed between different test conditions. For the jump tests, recovery was extended to approximately 2 min between conditions to minimize fatigue. In all the tests, data were acquired using a single Beyond Inertial wearable inertial measurement unit (IMU; Sensors Medica, Guidonia, Italy). The sensor measured 50 × 70 × 20 mm, weighed 35 g, and integrated a triaxial accelerometer (full-scale range: ±2 to ±16 g), a triaxial gyroscope (±2000°/s), and a triaxial magnetometer (±4800 µT). To reduce motion artefacts while maintaining participant comfort, the sensor was secured to the tested body segment using a semi-elastic belt with a magnetic mount. Data were transmitted via Bluetooth to a dedicated computer and processed at a sampling frequency of 500 Hz using the manufacturer’s software. Sensor placement was standardized according to the specific task under investigation. Prior to each dynamic task, a short static baseline acquisition was recorded in the starting position to define the reference orientation of the sensor.

#### 2.2.1. Balance Assessment

Postural control was assessed under four conditions: bipodalic stance with eyes open, bipodalic stance with eyes closed, right single-leg stance, and left single-leg stance. In the bipodalic conditions, athletes stood upright with feet parallel, arms relaxed alongside the body, and body weight evenly distributed on both lower limbs. In the single-leg conditions, participants maintained an upright posture while standing on one limb, with the contralateral limb elevated without ground contact. For the tasks performed with open eyes, participants were asked to fixate on a visual target positioned on a wall 5 m away and adjusted to each participant’s eye level.

Each trial lasted 30 s, and three valid repetitions were collected for each condition. Postural stability was quantified using IMU sensor, securely attached to the participant’s lower back at the L4 vertebral level to measure the linear acceleration and angular velocity of the pelvis. For each subject, the vertical distance from the center of the sensor to the ground was recorded prior to the test.

#### 2.2.2. Jump Performance

Explosive lower-limb performance was evaluated through the squat jump (SJ) and countermovement jump (CMJ). For both tests, participants started from an upright standing position with their feet approximately shoulder-width apart and their arms free to move during the jump.

In the SJ, athletes descended to a semi-squat position with approximately 90° of knee flexion, maintained a brief isometric pause, and then performed a maximal vertical jump. This condition was used to assess concentric explosive force production without the contribution of the stretch–shortening cycle. In the CMJ, participants performed a rapid downward countermovement immediately followed by a maximal vertical jump, thus allowing evaluation of neuromuscular efficiency and stretch–shortening cycle utilization.

Three valid trials were recorded for each jump condition. Kinematic data were gathered using IMU sensor, securely attached to the participant’s lower back at the L4 vertebral level to capture the linear acceleration and angular velocity of the pelvis. It is worth noting that similar inertial systems positioned at the waist or lumbar region have previously shown acceptable-to-high validity for vertical jump assessment; for example, the Gyko inertial sensor showed excellent concurrent validity for CMJ and SJ height, with ICC values ranging from 0.81 to 0.87 when compared with a force plate and Optojump [16]. Given the high-impact nature of these dynamic tasks, the IMU accelerometer’s full-scale range was increased to ±16 g.

#### 2.2.3. Upper-Limb Mobility Assessment

Shoulder mobility was assessed by means of active flexion and extension tasks performed separately with the right and left arms. Tests were conducted in the standing position without external loads. Participants were instructed to perform each movement voluntarily through the greatest possible range of motion while minimizing trunk compensations and accessory movements. The movement started from a neutral standing position with arms resting alongside the torso. Three complete flexion–extension trials were collected for each limb. For these three movements, an IMU sensor was positioned on the arm, 15 cm distal to the shoulder’s center of rotation.

### 2.3. Data Processing and Analysis

Data recorded during the initial static acquisition were used to align the sensor axes with a global reference frame. Subsequently, the raw linear acceleration and angular velocity signals acquired by the sensors were processed using a sensor-fusion approach to estimate the three-dimensional orientation of the sensor throughout each task [17].

**Figure 1:**
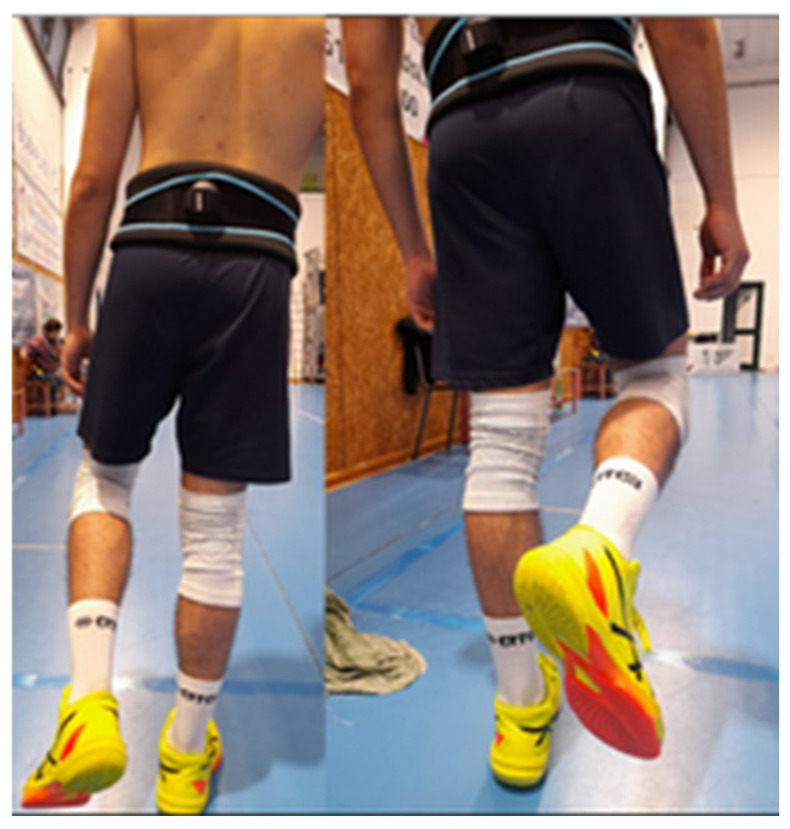
Balance assessment setup: subject wearing an elastic band securing the IMU sensor to the pelvis. The images illustrate the single-leg stance tasks.

For the balance assessment (Figure 1), postural sway was quantified by projecting the sensor orientation onto the horizontal plane to obtain an estimate of body sway trajectories in the anteroposterior and mediolateral directions. Specifically, the projection was obtained through a geometric procedure based on the initial sensor-to-floor height, measured with a tape measure prior to the test. The resulting two-dimensional coordinate was considered a reliable estimate of the whole-body Center of Mass (CoM), enabling the extraction of its anteroposterior (AP) and mediolateral (ML) sway trajectories [17,18]. Standard posturographic indices were then computed to assess postural control. On the basis of these trajectories, standard indices were computed following the equation reported in [19]: (i) total path length (PL); (ii) 95% confidence ellipse area (EA); (iii) directional sway lengths in the AP (SP_AP_) and (iv) ML (SP_ML_) axes. Specifically, PL denotes the total path length of the CoM, computed as the sum of the Euclidean distances between successive CoM points. EA was defined as the area of the 95% bivariate confidence ellipse, which statistically encompasses about 95% of the recorded CoM trajectory points. All the parameters were averaged across the three repetitions, independently for each stability task.

Concerning the jump performance analysis, the vertical linear acceleration recorded through IMU was numerically integrated to obtain vertical velocity, allowing for an accurate identification of the eccentric and concentric phases of the jumps. The eccentric phase was considered as the interval from the onset of the downward movement to the moment when velocity reached zero, corresponding to the transition point at which the subject reversed direction to push upward. The concentric phase was defined as the subsequent interval, starting from this zero-velocity point and continuing until take-off. Subsequently, the reported parameters were computed:

- Jump height—JH [m]: This is the maximum vertical displacement of the body’s center of mass achieved during the jump, typically estimated from flight time and used as a key indicator of explosive lower-limb performance.
- Flight time—FT [s]: This is defined as the duration the subject remains airborne from take-off to landing, reflecting jump performance.
- Concentric phase duration—CP [s]: This is the time interval during which the subject produces upward force, from the end of the eccentric phase to take-off.
- Eccentric phase duration—EP [s]: This is the time interval from the onset of the downward movement to the instant when velocity reaches zero, marking the transition from descent to upward propulsion. This parameter was computed only for CMJ.
- Eccentric-to-concentric ratio—ECR: This is the ratio between the duration of the eccentric phase and that of the concentric phase, providing insight into movement strategy and stretch–shortening cycle efficiency. This parameter was computed only for CMJ.
- Maximum velocity—MV [m/s]: This is defined as the highest vertical velocity reached during the concentric phase.
- Trunk inclination—TI [°]: This is the angle of the torso relative to the vertical axis during the movement, indicating posture and technique.

Each parameter was then averaged across the three repetitions, independently for each jump type and each participant.

**Figure 2:**
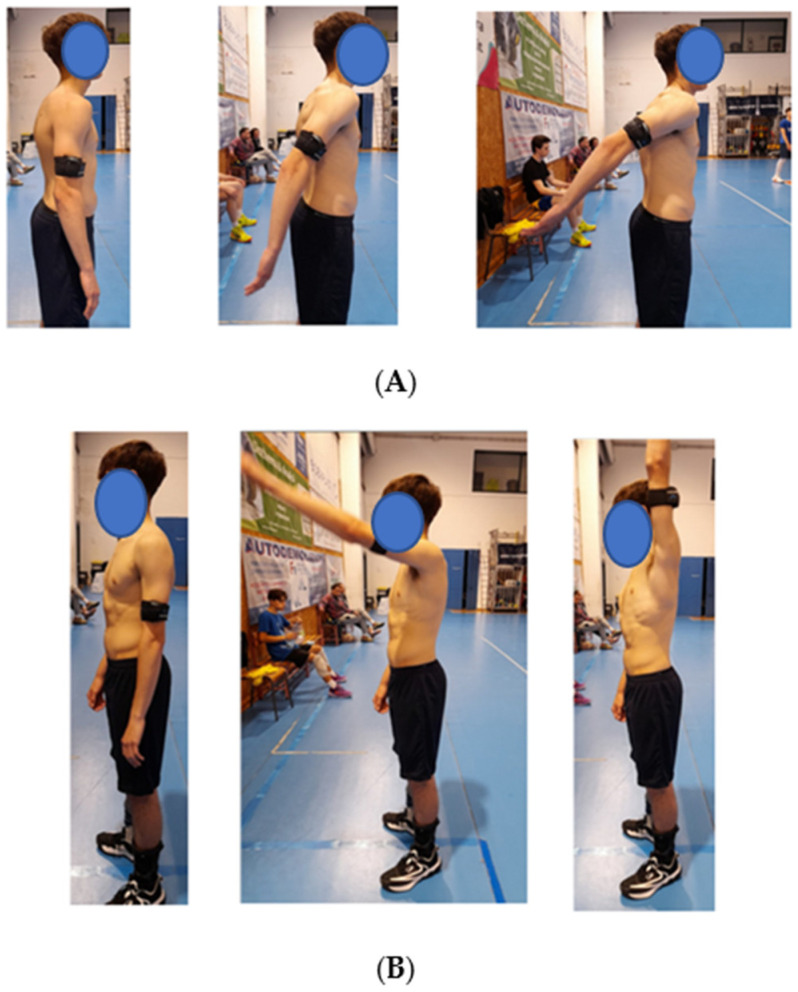
Upper-limb mobility assessment: subject wearing an elastic band securing the IMU sensor to the arm during (**A**) shoulder extension and (**B**) shoulder flexion.

Finally, moving to the mobility assessment (Figure 2), the maximum angular displacement along the examined axis was computed and denoted as θ per each movement and each side (θ_r_flex_, θ_r_ext_, θ_l_flex_ and θ_l_ext_). Specifically, θ was computed as the maximum differences between the angle measured during the execution of the movement and the baseline recorded during the starting resting position. The average value of θ was computed considering the three repetitions, independently for each movement (flexion and extension), each side and each participant. In the present field-based protocol, flexion and extension were selected because they could be assessed more reliably and with fewer compensatory strategies than rotational movements under ecological testing conditions.

### 2.4. Statistical Analysis

Raw data were exported and processed in MATLAB (MathWorks, Inc., Natick, MA, USA, version 2022a). Descriptive statistics were calculated for all variables. Normality was assessed using the Shapiro–Wilk test, whereas homogeneity of variances was verified using Levene’s test. All parameters were found to be normally distributed. Differences between playing positions were evaluated using one-way ANOVA. In cases where significant differences were identified, post hoc pairwise comparisons were conducted using Bonferroni for multiple comparisons. Statistical significance was set at *p* < 0.05. Statistical power was estimated using G*Power software (version 3.1.9.6), indicating an overall power of 0.82 assuming a medium effect size (f = 0.25) and an alpha level of 0.05 [20,21].

## 3. Results

Mean values and standard deviations, together with statistical differences, of the indices related to the balance assessment are reported in Table 2.

By analyzing the results reported in Table 2, significant role-related differences emerged across all balance conditions. In the bipodalic eyes-open (OE) condition, the role effect showed a mixed pattern: HIT displayed the largest ellipse area (8.5 ± 2.1 cm^2^), whereas SET showed the highest path-related values, including LP (10.1 ± 1.1 cm) and SP_ML_ (8.1 ± 1.4 cm). Post hoc comparisons indicated that HIT showed significantly greater EA than all the other roles, whereas SET significantly exceeded the other groups in the main path-related variables. By contrast, MB generally showed the most contained oscillations. In the bipodalic eyes-closed (CE) condition, the clearest pattern was observed for HIT, who consistently showed the highest values across all stabilometric variables, including EA (11.2 ± 3.1 cm^2^) and LP (36.1 ± 3.3 cm), whereas the remaining roles showed markedly lower and relatively clustered values. Post hoc analysis showed that HIT significantly exceeded all the other roles in this condition. The single-leg dominant-leg (D) condition further amplified between-role differences. In this task, OH showed the highest values for all stabilometric parameters, including EA (60.3 ± 6.9 cm^2^), LP (30.5 ± 1.1 cm), SP_ML_ (18.5 ± 2.2 cm), and SP_AP_ (21.2 ± 1.9 cm), significantly exceeding the other groups. A different ranking emerged in the single-leg non-dominant-leg (ND) condition, where LIB, OH, and HIT showed the highest ellipse area values, whereas LIB also showed the highest LP, SP_ML_, and SP_AP_, indicating a more distinct postural profile in this task.

Moving to the jump performance, Table 3 reports the mean and standard deviation of each parameter, divided per each task and each role. The same table also shows statistical differences.

Role-related differences were also evident in both SJ and CMJ. In the SJ, the clearest pattern concerned overall vertical performance. MB showed the highest jump height (44.7 ± 1.2 cm), significantly exceeding all the other roles, whereas LIB (32.1 ± 1.3 cm) and SET (33.3 ± 1.1 cm) showed the lowest values. Post hoc analysis confirmed that MB significantly differed from all the other groups for JH, while LIB and SET showed lower values than OH and HIT. OH and HIT displayed intermediate values. A similar trend was observed for FT and VM, with MB again showing the most favorable profile. A different pattern emerged for movement timing. In the SJ, the longest CP was observed in HIT (0.73 ± 0.12 s), whereas MB and OH showed the shortest concentric times. TI also differentiated the groups, with SET and HIT showing the highest values and OH the lowest. In the CMJ, the overall hierarchy remained similar, with MB again showing the highest JH, FT, and VM, while LIB and SET remained at the lower end of the distribution. Again, post hoc comparisons indicated that MB significantly exceeded the other roles for the main jump-output variables. More specific role-related differences emerged for temporal variables, with OH showing the longest EP (0.83 ± 0.15 s) and SET the longest CP (0.46 ± 0.06 s). In addition, OH showed the lowest TI, whereas LIB, SET, and HIT showed the highest values.

**Figure 3:**
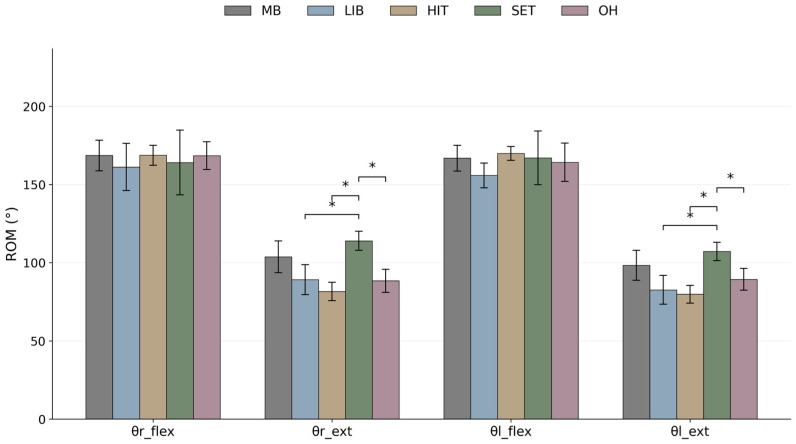
Mean value and standard deviation of mobility parameters for each movement and each role. * indicates statistical differences.

Finally, the results of the mobility are reported in Figure 3, with the statistical difference highlighted. Upper-limb mobility showed a more selective role effect. Flexion values were relatively homogeneous across roles, with no clear between-group differences in either θ_r_flex_ or θ_l_flex_. By contrast, extension showed a clearer positional pattern. In θ_r_ext_, SET showed the highest ROM (113.9 ± 6.1°), exceeding LIB, HIT, and OH, whereas MB showed an intermediate profile (103.8 ± 10.2°) and did not differ from setters. A similar distribution was found for θ_l_ext_, with SET again showing the highest ROM (107.2 ± 5.9°) and MB remaining the closest group. Post hoc analysis confirmed that SET significantly exceeded LIB, HIT, and OH in both extension tasks, whereas no significant difference was observed between SET and MB. Overall, these findings indicate that extension, rather than flexion, was the mobility parameter most sensitive to positional specialization.

**Figure 4:**
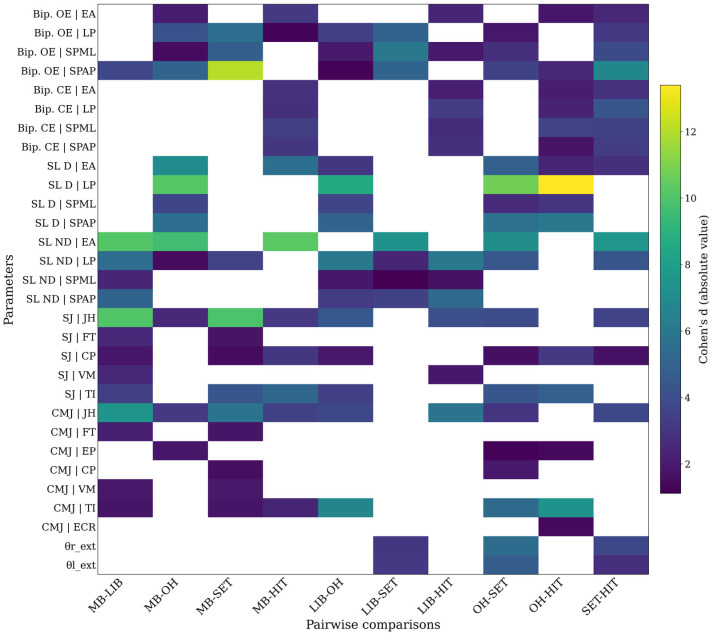
Heat map of Cohen’s *d* for pairwise comparisons.

As a summary, a total of 32 one-way ANOVAs were performed across balance, jump, and mobility variables. Among these, 30 reached statistical significance and were followed by Bonferroni-adjusted post hoc pairwise comparisons. Given the five-group design, this corresponded to up to 10 pairwise contrasts per significant variable, for a maximum of 300 post hoc comparisons. To complement *p*-values reported in the previous tables and figure, effect sizes were reported as eta squared (η^2^) for ANOVAs and Cohen’s *d* for pairwise comparisons in Table 4 and Figure 4, respectively. Effect size values indicated that several of the observed differences were not only statistically significant, but also practically meaningful.

## 4. Discussion

### 4.1. Is Balance Performance Role-Related?

The present results indicate that postural control differed across playing roles, although the expression of these differences depended on task condition. This overall pattern is in line with previous evidence suggesting that volleyball players show sport-specific balance characteristics rather than a uniform static stability profile [12,13], and more generally with the view that balance behavior in athletes may reflect the demands typically associated with the practiced sport [22,23,24]. In the bipodalic tasks, role-related differences were already detectable in relatively simple stance conditions. In the eyes-open condition, HIT showed the largest ellipse area, whereas SET showed the highest path-related values. This pattern is of interest from a role perspective, because outside hitters are frequently involved in transitions between reception, approach, attack, landing, and repositioning, while setters are repeatedly required to organize body orientation and ball-related positioning under time constraints [25]. The eyes-closed condition showed a clearer pattern, with HIT presenting the highest values across all stabilometric parameters. These findings do not directly identify the mechanisms underlying the observed sway differences, but they suggest that the static postural profile associated with hitters may be more distinct than that of the other roles under reduced visual information. This observation broadly agrees with the volleyball literature showing that postural behavior may vary according to the specific functional demands placed on players [26].

A stronger role effect emerged in the single-leg tasks. On the dominant side, OH showed the highest values for all stabilometric variables, whereas in the non-dominant condition, LIB was most frequently involved in significant contrasts, particularly for total and directional sway. These findings are compatible with the asymmetrical nature of several volleyball actions and with the fact that different roles are repeatedly exposed to different unilateral demands. In particular, opposite hitters are more frequently associated with repeated attack and landing actions close to the net [5], whereas liberos are more often involved in defensive repositioning, low-body support, and rapid directional adjustments [9]. The present results do not demonstrate that such task exposure determines the observed balance profile, but they do suggest that the postural behavior of OH and LIB may be more role-specific in unilateral conditions than in bipodalic stance. In this sense, the present findings are partially consistent with Agostini et al. [12], who proposed that functional screening may reveal role-related features in volleyball players, even though the number of significant contrasts in their study was more limited. Finally, the relatively contained sway shown by MB in some of the simpler conditions may also be read within the broader positional profile already described in volleyball. Previous studies have shown that middle blockers often differ from other roles in anthropometric and functional characteristics [7]. In the present sample, their simpler-task postural profile appeared less dispersed than that of other groups, particularly in bipodalic stance. This does not necessarily indicate superior balance in an absolute sense, but it supports the idea that role-related differences in volleyball may also extend to static postural organization.

### 4.2. Is Jump Performance Role-Related?

The jump results showed the clearest role-related pattern in the present study. In both SJ and CMJ, MB displayed the most favorable vertical profile, with the highest jump height, flight time, and maximum velocity, whereas LIB and SET generally occupied the lower end of the distribution. This result is in substantial agreement with previous volleyball literature; in fact, Lima et al. [11] reported position-specific jump profiles in official matches, Mielgo-Ayuso et al. [27] found lower jump-related performance in liberos, and Sheppard et al. [25] described middle players as the role most exposed to blocking and spike-jump demands. Similarly, Palao et al. [7] reported positional differences in physical and anthropometric characteristics that tend to favor net-dominant roles. Therefore, the present findings fit well with the broader literature indicating that MB and, more generally, front-row roles are associated with stronger vertical-performance profiles.

The current results also show that role-related differences were not limited to jump output. In the SJ, HIT showed the longest concentric phase, whereas in the CMJ, OH showed the longest eccentric phase and SET the longest concentric phase. This suggests that different roles may also be associated with different temporal organizations of force production. From a role perspective, this appears relevant because outside hitters and opposite hitters are more frequently linked to repeated attack-related jump demands, whereas setters are commonly involved in frequent jump-setting actions that are repeated but not necessarily maximal in height [6,28]. In this respect, the present findings are broadly consistent with previous work showing that jump-related performance in volleyball depends not only on jump height, but also on force–time and force–velocity characteristics [29,30,31]. The higher vertical values observed in MB are also coherent with the literature describing this role as one of the most specialized for repeated net actions. Earlier studies have shown that middle blockers are generally among the tallest players and are frequently characterized by favorable conditions for blocking and quick attack actions [25,32]. In the present data, this broader positional profile was accompanied by the best performance in both jump tasks. At the same time, the lower outputs observed in SET and LIB align with the different competitive functions of these roles since setters tend to accumulate a high number of jumps that are more closely linked to ball organization than to maximal propulsion [25,28], whereas liberos are less directly involved in repeated maximal net-jump actions [9,27]. The present design does not establish whether these differences derive from training exposure, role selection, or both, but it does indicate that jump performance remains one of the most clearly role-related functional domains in volleyball, confirming [33,34].

A further point of interest concerns trunk inclination. In the present study, SET and HIT showed higher values, whereas OH showed lower values, especially in the CMJ. This finding may suggest that roles differ not only in jump level, but also in how they organize body segments during propulsion. This observation is broadly compatible with the wider biomechanics literature showing that trunk position influences vertical-jump mechanics [35,36], and it adds a further element to the role-specific interpretation of jumping behavior.

### 4.3. Is the Mobility of Upper Limb Role-Related?

The mobility findings suggest that upper-limb ROM in volleyball may be only partly role-dependent, with the clearest positional difference emerging in extension rather than flexion. The absence of marked between-role differences in θ_r_flex_ and θ_l_flex_ may indicate that flexion represents a more general functional requirement shared across positions. This appears coherent with the technical structure of volleyball, since overhead arm elevation is involved, although with different purposes and frequencies, in serving, attacking, blocking, setting, and some defensive overhead actions [4]. Therefore, a relatively homogeneous flexion profile across roles may be viewed as compatible with a common exposure to repeated overhead movements during training and competition [15]. A different pattern emerged for extension, where setters showed the most distinctive profile. In both right and left extension, setters presented higher ROM values than liberos, outside hitters, and opposite hitters, while not differing from middle blockers. From a role-related perspective, this result is compatible with the repeated overhead positioning and upper-limb organization required in setting actions. Setters are continuously asked to align the trunk and upper limbs with the ball trajectory and to perform precise overhead contacts under variable spatial and temporal constraints [8]. In this sense, a greater extension ROM may fit with the need for repeated arm positioning above and slightly behind the frontal plane, where shoulder mobility and segmental coordination contribute to movement efficiency and ball control [4,15]. The fact that MB did not differ substantially from setters is also of interest. Although the technical goals of the two roles are different, both are repeatedly exposed to overhead actions near the net, especially during blocking and rapid arm elevation [4]. This shared exposure may be compatible with the relatively similar extension values observed in these two groups. By contrast, the lower extension values observed in LIB, HIT, and OH may be viewed in relation to the different technical priorities of these roles. Liberos are more frequently engaged in low defensive positions, reception, and floor-related reorganization [9], whereas outside hitters and opposite hitters are more strongly characterized by high-velocity attacking actions and repeated powerful arm swings [10]. Within this context, the present data may suggest that extension ROM is not expressed uniformly across roles, but tends to reflect, at least in part, the different upper-limb demands associated with volleyball positioning and task specialization.

At the same time, direct comparison with the literature remains limited, because most available studies have examined upper-limb function through technical gesture analysis rather than through simple field-based ROM profiling across roles [15]. For this reason, the present findings are better interpreted as preliminary evidence that some mobility characteristics, particularly extension, may vary across playing positions in a way that is broadly consistent with role-specific volleyball demands.

### 4.4. Practical Implications and Limitations

The present findings support a role-specific functional interpretation of volleyball performance. From an applied perspective, the main practical implications are as follows:

- Middle blockers and opposite hitters showed the most favorable vertical performance profile, suggesting that explosive lower-limb capacities are particularly relevant for net-dominant roles.
- Outside hitters combined greater postural sway with longer concentric execution, indicating a functional profile that may require careful monitoring of both stability and force-production strategy.
- Setters showed lower jump output but greater upper-limb extension ROM, together with distinctive balance behavior in bipodalic tasks, suggesting a more coordination- and control-oriented functional profile.
- Liberos stood out especially in the non-dominant single-leg condition, supporting the idea that defensive roles may rely on specific postural and asymmetry-related adaptations.

Overall, these data suggest that balance, jump mechanics, and upper-limb mobility should be assessed together, as their combined evaluation may help practitioners better characterize positional demands and individualize monitoring strategies across the season.

The present findings should be interpreted in light of some limitations. First, the relatively small sample size within each playing role may limit the generalizability of the results. In addition, although all participants were competitive players with several years of practice and regular exposure to team training and official matches, a more detailed quantification of individual training history was not available; therefore, differences in cumulative volleyball experience may have partly influenced the observed functional profiles. Another important limitation is that the protocol did not directly assess volleyball-specific technical gestures, but rather selected functional capacities underlying performance, namely postural control, jump ability, and upper-limb mobility. As a consequence, the results should be interpreted as role-specific functional profiles rather than as direct biomechanical descriptions of technical execution.

A further limitation concerns the potential influence of anthropometric characteristics such as height and body mass. Although descriptive anthropometric data were reported to play a role in improving transparency, these variables are known to differ across volleyball positions and may still have contributed, at least in part, to some of the observed differences, particularly in jump and balance performance. At the same time, in volleyball, the anthropometric profile is closely intertwined with positional specialization and therefore cannot be considered a simple nuisance factor. For this reason, the present findings are better interpreted as integrated role-specific profiles shaped by both morphology and task-specific functional characteristics, rather than as role effects entirely independent of body-size differences. Methodological limitations should also be acknowledged. Jump height was estimated from a single pelvis-mounted IMU using a flight-time-based approach, and no device-specific validation against a force plate or optical system was performed for the Beyond sensor and algorithm. Although similar waist- and lumbar-mounted inertial systems have shown acceptable validity for vertical jump assessment, the present jump values should be interpreted primarily for between-group comparisons within the same experimental setup rather than as absolute criterion measures. Moreover, because trunk inclination differed across roles, a systematic bias in center-of-mass estimation cannot be excluded. Finally, upper-limb mobility assessment was limited to flexion and extension, whereas internal and external rotation were not included. This choice was made to preserve the simplicity and field applicability of the protocol, but it reduced the completeness of the shoulder functional profile.

## 5. Conclusions

This study showed that volleyball players display role-specific functional profiles in postural control, jump performance, and upper-limb mobility. In particular, middle blockers showed the most favorable jump profile, setters were characterized by greater upper-limb extension ROM, and balance performance varied according to both task condition and playing role. These findings support the use of an integrated functional assessment to better describe positional demands in competitive volleyball. It is important to note that the role-related functional profiles observed in the present study may reflect the combined contribution of training specialization, anthropometric selection, and task-specific exposure. Longitudinal studies will be needed to better clarify the relative influence of these factors over time. Future studies should confirm these findings in larger samples, include female athletes and different competitive levels, and further investigate the contribution of anthropometric, neuromuscular, and technical factors to role-specific performance.

## Figures and Tables

**Table 1:** Anthropometric data of the participants according to the playing positions.

Role	Age (Years)	Height (m)	Body Mass (kg)
MB	21.2 ± 3.0	1.95 ± 0.05	79.8 ± 6.4
LIB	19.8 ± 2.8	1.76 ± 0.05	65.2 ± 5.1
OH	20.9 ± 3.1	1.91 ± 0.04	76.5 ± 5.8
SET	20.1 ± 2.9	1.83 ± 0.05	69.4 ± 5.3
HIT	20.4 ± 3.0	1.88 ± 0.06	72.8 ± 5.9

**Table 2:** Mean (SD) of indices related to the balance assessment. D and ND stands for dominant and non-dominant. Superscripts indicate statistical differences among groups, where * indicates differences with all the other groups.

Task	Index	MB	LIB	OH	SET	HIT
**Bipodalic OE**	EA (cm^2^)	3.3 (0.9) ^OH,HIT^	4.5 (1.1) ^HIT^	5.4 (1.1) ^MB,HIT^	4.6 (0.5) ^HIT^	8.5 (2.1) *
	LP (cm)	2.5 (1.6) ^HIT,OH,SET^	4.3 (1.2) ^OH,SET^	8.1 (1.0) ^MB, LIB,SET^	10.1 (1.1) *	4.8 (2.1) ^MB,SET^
	SP_ML_ (cm)	2.2 (1.0) ^SET^	1.9 (0.4) ^SET^	4.1 (1.5) ^MB, LIB,SET^	8.1 (1.4) *	3.4 (1.0) ^LIB,SET^
	SP_AP_ (cm)	1.7 (0.3) ^LIB,OH,SET^	4.7 (1.1) ^MB,OH,SET^	6.1 (1.2) *	9.8 (0.9) *	3.3 (1.0) ^OH,SET^
**Bipodalic CE**	EA (cm^2^)	4.5 (1.1) ^HIT^	5.5 (2.1) ^HIT^	5.6 (2.1) ^HIT^	3.8 (1.9) ^HIT^	11.2 (3.1) *
	LP (cm)	25.2 (4.4) ^HIT^	25.7 (3.1) ^HIT^	27.4 (4.3) ^HIT^	24.1 (2.0) ^HIT^	36.1 (3.3) *
	SP_ML_ (cm)	14.7 (2.1) ^HIT^	14.7 (2.8) ^HIT^	14.5 (2.1) ^HIT^	14.6 (2.1) ^HIT^	20.6 (1.2) *
	SP_AP_ (cm)	17.2 (2.2) ^HIT^	18.1 (2.1) ^HIT^	19.8 (3.3) ^HIT^	16.2 (2.3) ^HIT^	25.4 (3.1) *
**Single-leg D**	EA (cm^2^)	21.9 (3.3) ^OH^	40.2 (6.2) ^OH^	60.3 (6.9) *	33.9 (3.3) ^OH^	45.9 (5.1) ^MB,SET,OH^
	LP (cm)	18.8 (1.2) ^OH^	20.1 (1.3) ^OH^	30.5 (1.1) *	19.7 (0.9) ^OH^	19.7 (0.3) ^OH^
	SP_ML_ (cm)	10.6 (2.1) ^OH^	12.0 (1.2) ^OH^	18.5 (2.2) *	12.9 (2.1) ^OH^	12.4 (1.8) ^OH^
	SP_AP_ (cm)	12.4 (1.2) ^OH^	13.3 (1.2) ^OH^	21.2 (1.9) *	11.9 (1.3) ^OH^	11.4 (1.3) ^OH^
**Single-leg ND**	EA (cm^2^)	21.2 (1.4) ^LIB,OH,HIT^	39.8 (2.2) ^MB,SET^	40.1 (2.4) ^MB,SET^	23.2 (2.3) ^LIB,OH,HIT^	40.1 (2.2) ^MB, SET^
	LP (cm)	14.1 (1.5) ^SET,OH,LIB^	22.9 (1.7) *	11.4 (2.1) ^MB,SET,LIB^	19.2 (1.3) *	12.3 (1.8) ^SET,LIB^
	SP_ML_ (cm)	8.6 (1.1) ^LIB^	12.1 (1.7) *	9.5 (0.9) ^LIB^	10.5 (1.1) ^LIB^	9.5 (1.4) ^LIB^
	SP_AP_ (cm)	9.9 (0.4) ^LIB^	14.8 (1.3) *	10.7 (1.2) ^LIB^	10.6 (1.1) ^LIB^	7.9 (1.3) ^LIB^

**Table 3:** Mean (SD) of indices related to the jump performance for both squat jump (SJ) and countermovement jump (CMJ). Superscript indicates statistical differences among groups, where * indicates differences with all the other groups.

Task	Index	MB	LIB	OH	SET	HIT
**SJ**	JH (cm)	44.7 (1.2) *	32.1 (1.3) ^OH,HIT,MB^	40.2 (2.2) ^SET,LIB,MB^	33.3 (1.1) ^OH,HIT,MB^	39.3 (2.1) ^SET,LIB,MB^
	FT (s)	0.60 (0.04) ^LIB,SET^	0.51 (0.03) ^MB^	0.56 (0.03)	0.53 (0.04) ^MB^	0.55 (0.02)
	CP (s)	0.39 (0.10) ^LIB,SET,HIT^	0.63 (0.15) ^MB,OH^	0.38 (0.10) ^LIB,SET,HIT^	0.54 (0.10) ^MB,OH^	0.73 (0.12) ^MB,OH,SET^
	VM (m/s)	3.0 (0.2) ^LIB^	2.6 (0.1) ^MB,HIT^	2.8 (0.2)	2.7 (0.3)	2.9 (0.2) ^LIB^
	TI (°)	25.8 (3.3) ^SET,HIT,LIB^	34.4 (1.3) *	23.2 (4.4) ^SET,HIT,LIB^	40.1 (3.2) ^LIB,MB,OH^	40.1 (2.1) ^LIB,MB,OH^
**CMJ**	JH (cm)	47.4 (2.2) *	34.1 (1.2) ^MB,OH,HIT^	40.6 (2.1) ^MB,LIB,SET^	33.9 (2.4)^MB,OH,HIT^	41.1 (1.2) ^MB,LIB,SET^
	FT (s)	0.63 (0.05) ^LIB,SET^	0.52 (0.05) ^MB^	0.57 (0.04)	0.52 (0.07) ^MB^	0.57 (0.06)
	EP (s)	0.58 (0.12) ^OH^	0.67 (0.11)	0.83 (0.15) ^MB,SET,HIT^	0.63 (0.17) ^OH^	0.60 (0.18) ^OH^
	CP (s)	0.32 (0.11) ^SET^	0.35 (0.12)	0.32 (0.08) ^SET^	0.46 (0.06) ^MB,OH^	0.38 (0.11)
	VM (m/s)	3.2 (0.2) ^LIB,SET^	2.7 (0.3) ^MB^	2.9 (0.1)	2.7 (0.3) ^MB^	2.9 (0.3)
	TI (°)	36.7 (2.2) ^LIB,SET,HIT^	40.4 (1.9) ^MB,OH^	29.2 (1.4) ^LIB,SET,HIT^	41.4 (2.9) ^MB,OH^	41.6 (1.9) ^MB,OH^
	ECR	2.1 (0.4)	2.3 (0.7)	2.8 (0.9) ^HIT^	2.5 (0.8)	1.7 (0.6) ^OH^

**Table 4:** Effect-size (η^2^) for all the ANOVA tests.

Test Family	Task	Variable	F (df = 4,45)	Effect-Size (η^2^)
Balance	Bipodalic CE	EA	24.35	0.622
		LP	44.48	0.626
		SP_AP_	104.52	0.627
		SP_ML_	48.69	0.585
	Bipodalic OE	EA	18.53	0.684
		LP	18.82	0.798
		SP_AP_	18.88	0.903
		SP_ML_	15.87	0.812
	Single-leg D	EA	75.72	0.871
		LP	229.94	0.953
		SP_AP_	84.86	0.881
		SP_ML_	25.04	0.691
	Single-leg ND	EA	210.77	0.949
		LP	84.36	0.881
		SP_AP_	50.91	0.819
		SP_ML_	11.00	0.494
Jump	CMJ	CP	3.48	0.236
		ECR	3.50	0.237
		EP	4.52	0.287
		FT	6.85	0.379
		JH	90.71	0.887
		TI	61.09	0.844
		VM	6.56	0.368
	SJ	CP	17.24	0.605
		FT	10.65	0.486
		JH	100.53	0.899
		TI	66.16	0.856
		VM	5.68	0.336
Mobility	Left extension	θ_l_ext_	21.95	0.661
	Left flexion	θ_l_flex_	2.42	0.177
	Right extension	θ_r_ext_	26.90	0.705
	Right flexion	θ_r_flex_	0.66	0.056

## Data Availability

Data are available upon request to the corresponding editor.
